# RELATIONSHIP-CENTERED CARE: ADULT DAY CARE FOR PERSONS LIVING WITH DEMENTIA AND THE SENSES FRAMEWORK

**DOI:** 10.1093/geroni/igz038.282

**Published:** 2019-11-08

**Authors:** Samantha Woog, Eleanor McConnell, Deborah Gold, Kirsten Corazzini

**Affiliations:** 1 Duke University, Durham, North Carolina, United States; 2 Duke, Hillsborough, North Carolina, United States

## Abstract

Relationship-centered dementia care (RCDC) has been related to improved quality of residential long-term care for persons living with dementia (PLWD). The senses framework supports accomplishing RCDC, whereby PLWD meet fundamental needs or senses through caregiving relationships. This study explored the application of the senses framework to a non-residential, long-term care setting, and included relationships across formal and informal caregivers. The study design is a qualitative, descriptive study of PLWD (N=3), with matched formal (N=3) and informal (N=3) caregivers in one adult day care setting in North Carolina. Semi-structured individual interviews explored each of the six senses of security, belonging, continuity, purpose, achievement, and significance. Interviews were analyzed using both inductive and deductive thematic analysis. Themes elucidate convergence and divergence of how senses are met or not met across triads of caregiving relationships. Findings inform our understanding of how to integrate the larger social network of PLWD for relationship-centered care.

